# Successful FUT hair restoration following Mohs surgery with allograft repair

**DOI:** 10.1016/j.jdcr.2026.05.026

**Published:** 2026-05-19

**Authors:** Chloe Fernandez, An Thien Nguyen, Shoshana Trudel, Nicole C. Cabbad, Leena Ramani, Adam Leavitt

**Affiliations:** a Department of Dermatology, Kansas City University – GME Consortium/Advanced Dermatology and Cosmetic Surgery Orlando Residency Program, Maitland, Florida; b Anne Burnett School of Medicine at Texas Christian University, Fort Worth, Texas; c University of Central Florida College of Medicine, Orlando, Florida; d Advanced Dermatology and Cosmetic Surgery Orlando Residency Program, Maitland, Florida; e Department of Dermatology and Cutaneous Surgery, University of Miami Miller School, Miami, Florida

**Keywords:** alopecia, basal cell carcinoma, biologic allograft, cicatricial alopecia, dehydrated human amnion/chorion membrane (dHACM), follicular unit transplantation, hair transplantation, Mohs micrographic surgery, scalp defect, scalp reconstruction

## Introduction

Basal cell carcinoma (BCC) is the most common skin cancer, with surgical excision as the standard treatment. Mohs micrographic surgery (MMS) offers lower recurrence rate and tissue-sparing advantages when compared to standard excision, and is the preferred method of treatment for high-risk BCCs.^1^ Large scalp defects present unique reconstructive challenges due to limited tissue elasticity and cosmetic concerns in preserving hair-bearing tissue.^2^ The authors present a large occipital scalp BCC treated with MMS, followed by placement of dehydrated human amnion/chorion membrane (dHACM) placental tissue allograft for healing, and subsequent cosmetic hair transplantation.

## Case report

**Fig 1:**
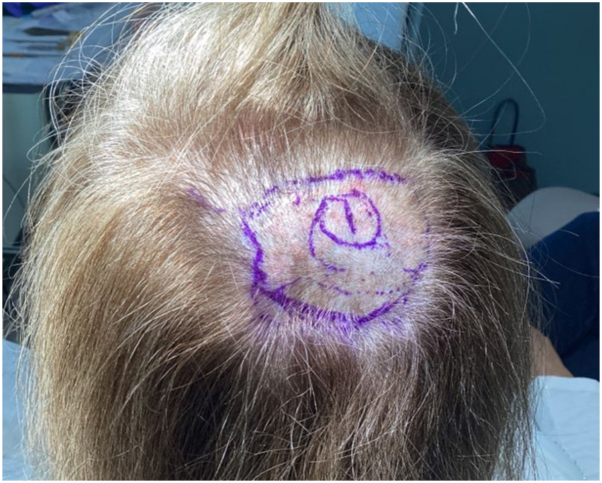
Pre-operative image of the BCC on the occipital scalp marked prior to MMS.

A 68-year-old woman with a prior history of BCC presented to her dermatologist with a pruritic 2-cm papule on the right superior occipital scalp. The lesion was treated with intralesional triamcinolone, topical clobetasol solution, and mupirocin ointment. She returned 6 m later without improvement. A shave biopsy was performed, and was diagnosed as a pigmented nodular BCC with involved margins. Treatment options were discussed, and the patient strongly preferred non procedural options due to cosmetic concerns in a hair-bearing area. A 6-week course of twice-daily topical 5% 5-fluorouracil therapy was initiated and was unsuccessful in shrinking the lesion. The patient was then referred to Mohs surgery for further management. Consultation with the Mohs surgeon revealed a lesion on the occipital scalp with a central well healed scar and peripheral abnormal appearing skin that appeared shiny and had arborizing telangiectases on dermoscopy suggestive of BCC and measuring 5-cm × 4-cm in size (Fig 1). Cosmetically acceptable reconstructive options were discussed, including healing by secondary intention, skin grafting, tissue expansion with rotational flap closure, and biologic allograft placement. The patient expressed a strong preference for maintaining hair-bearing scalp and natural hair orientation, as well as minimizing visible scarring. She declined tissue expansion due to its staged nature and temporary cranial distortion. Rotational flaps were avoided due to potential alteration in hair direction. The patient wished to avoid skin grafting to minimize donor-site morbidity and loss of native hair growth. With these preferences, she elected biologic allograft placement with gradual secondary healing, accepting a longer recovery to preserve scalp contour and allow for later hair transplantation if desired.

**Fig 2:**
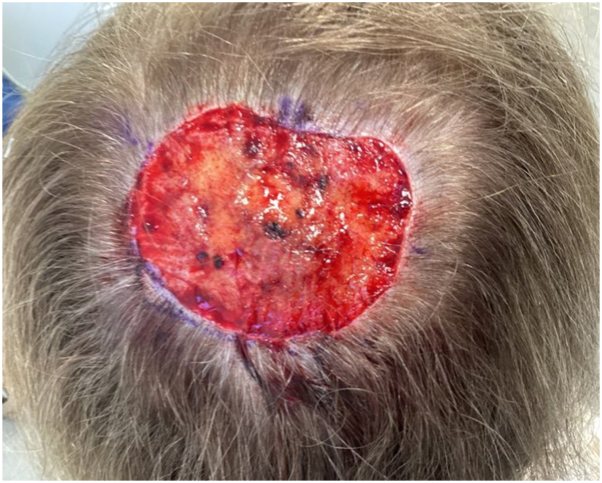
Post-stage 4 MMS defect measuring 6.9-cm × 5-cm prior to closure.

The lesion was completely cleared with 4 stages of MMS. Intraoperative histopathology revealed basaloid aggregates confined to the epidermis, without perineural invasion, consistent with her initial diagnosis of BCC, nodular pattern. The first stage of Mohs was taken full thickness to adipose tissue, where the clinical scar was. Residual superficial BCC remained at the peripheral margins, and the deep margin was clear. Three additional stages were necessary to clear superficial BCC. Care was made to excise the tissue above the level of the hair bulb/papilla where possible with the idea that some follicles would be able to regenerate. Average dermal hair papilla was located 4 mm below the scalp surface, visualized with 2.5 magnification loupes with a headlamp. Dissection was performed with visualization of the hair bulb. The final post-surgical defect measured 6.9-cm × 5-cm, extending into the adipose tissue, and was referred to plastic surgery for closure (Fig 2). Given the size and location, proximity to free margins, and previously discussed reconstructive goals, reconstruction was achieved using a dHACM placental tissue allograft (Epifix; MiMedx Group Inc, Marietta, GA) for coverage, which remained until complete granulation occurred.

**Fig 3:**
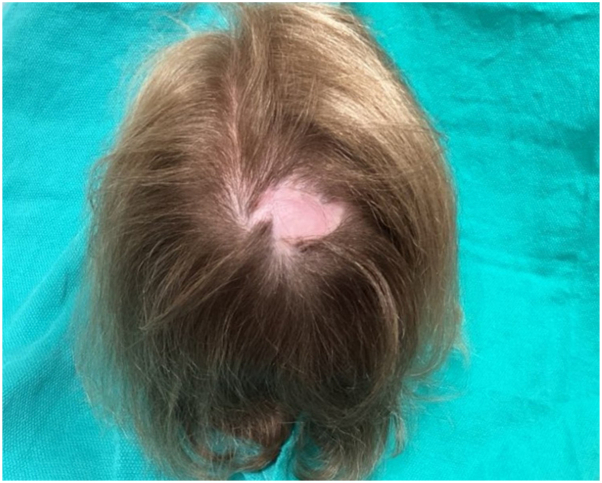
Healed surgical site demonstrating alopecic scar prior to hair transplantation.

**Fig 4:**
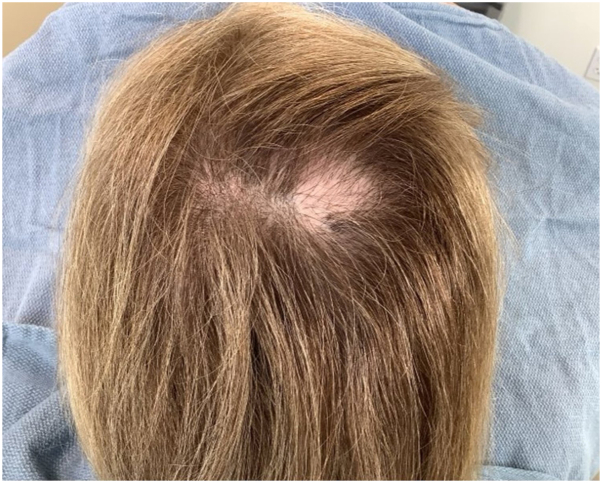
Post-follicular unit hair transplantation photograph showing improved coverage of the alopecic surgical scar.

**Fig 5:**
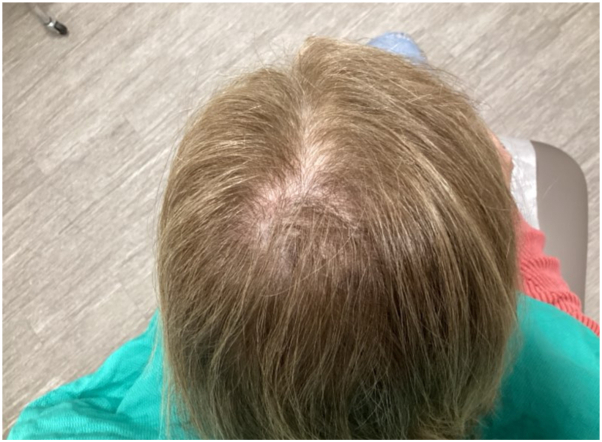
One year post-follicular unit hair transplantation photograph showing retained coverage of the alopecic surgical scar.

Due to cosmetic concerns regarding alopecia within the post-surgical scar (Fig 3), the patient began low dose oral 1.25 mg minoxidil daily therapy. The patient wore an artificial hair unit temporarily. Scar was digitally massaged frequently with the intent of softening the scar and increasing its vascularity. Sixteen months post-MMS, the patient underwent follicular unit hair transplantation. Six hundred and thirty-five grafts were harvested from a 1-cm × 10-cm donor mid occipital scalp and transplanted into the post-surgical scar, restoring hair density across the defect. Follow-up demonstrated excellent graft retention with new hair growth and no evidence of tumor recurrence (Fig 4). The graft survival/take rate is comparable to literature rates typically ranging 80% to 95%.^1^ One year post-transplant follow-up evinced graft maintenance, no tumor recurrence, and the patient is satisfied with current hair coverage and does not plan on follow-up procedures (Fig 5).

## Discussion

The scalp, a hair-bearing, sun-exposed surface, presents unique reconstructive challenges due to its convexity, inflexibility, and cosmetic importance. BCC surgical management requires intentional oncologic control and aesthetic outcomes.

Superficial BCCs may respond to topic therapies; in this case the 5-fluorouracil was insufficient for the more invasive components of the tumor. Imiquimod was offered to pre-treat the area, but the patient declined, wanting clear surgical margins. MMS was elected for maximal tissue preservation and its low recurrence rates—resulting in a 6.9 cm × 5 cm full-thickness scalp defect.^2^

Post-MMS scalp defects may be reconstructed using secondary intention healing, primary closure, negative pressure wound therapy, artificial dermis/skin grafts, local and regional flaps, tissue expansion, or free tissue transfer.^3^ Considering the size and location of the defect, the goal of enhancing tissue quality for possible future hair restoration, and the patient’s age and anticipated slower wound healing, a dHACM allograft was elected to facilitate granulation and re-epithelialization. dHACMs have demonstrated efficacy in chronic wounds, such as diabetic foot and venous stasis ulcers; yet, there is limited literature exploring dHACM in post-MMS defects.^4^ Its use in this case supported progressive wound healing, creating an optimized foundation for later reconstructive steps.

The patient was left with a stable, alopecic scar. Follicular unit hair transplantation was pursued due to her cosmetic concerns. This technique has demonstrated favorable outcomes in patients with cicatricial alopecia, reporting 12-month survival rates over 80%.^5^ Scar tissue is poorly vascularized, which limits the survival of grafted follicles. Graft density must be reduced centrally to compensate for hypoperfusion. This is the first known instance of hair transplantation into dHACM-treated tissue. Although dHACM does not directly promote follicular regeneration, its biologically active matrix supports angiogenesis and tissue remodeling. This fosters a favorable environment for subsequent follicular unit transplantation, thus combatting inherent limitations to transplantation procedures to scar tissue.^6^

The incorporation of follicular unit hair transplantation following complete oncologic and structural healing distinguishes this case. While stable cicatricial alopecia is often tolerated, the patient’s desire for hair restoration reflects emphasis on patient autonomy in quality of life. The level of Mohs dissection was shallow, which resulted in less scar tissue/contracture. There was less healing by secondary intention because of the Epifix, which resulted in softer, more pliable tissue. More grafts were placed towards the outer edge of the scar, where there is higher vascularization. Additional grafts were dispersed in healthy skin around the scar to improve density and camouflage. The successful transplantation, promoted by nutrient-dense dHACM allograft, suggests biologic dressings not only healing but also facilitating cosmetic reconstruction.

The multidisciplinary, staged approach, from oncologic clearance to biologic supported healing to elective aesthetic restoration, emphasizes the importance of individualized healthcare. The patient’s decision to accept a longer healing course to preserve scalp contour, support future hair restoration, minimize scarring, and avoid staged or donor-site procedures illustrates how reconstructive planning that aligns with patient preferences can achieve both oncologic safety and satisfying cosmetic outcomes.

## Conflicts of interest

None disclosed.
